# Kinetic change of serum carcinoembryonic antigen can early predict progression in patients with metastatic non-small cell lung cancer during maintenance therapy with bevacizumab plus pemetrexed

**DOI:** 10.18632/oncotarget.20456

**Published:** 2017-08-24

**Authors:** Zhang Nasha, Kong Li, Shi Fang, Jing Wang, Wang Haiyong, Yang Ming, Yu Jinming, Zhu Hui

**Affiliations:** ^1^ Cheeloo College of Medicine, Shandong University, Jinan, China; ^2^ Department of Radiation Oncology, Shandong Cancer Hospital and Institute, Shandong Cancer Hospital Affiliated to Shandong University, Jinan, China; ^3^ Shandong Academy of Medical Sciences, Jinan, China

**Keywords:** CEA kinetic change, progression, metastatic NSCLC, maintenance therapy, bevacizumab

## Abstract

In this retrospective study, we investigated whether the kinetic change of serum carcinoembryonic antigen (CEA) levels can be an early indicator for the progression in metastatic non-small cell lung cancer (NSCLC) patients during maintenance therapy with bevacizumab plus pemetrexed. Ten patients diagnosed with metastatic lung adenocarcinoma who received a first-line therapy including bevacizumab-based chemotherapy and a following maintenance therapy including bevacizumab plus pemetrexed from June 2015 to October 2016 were recruited in this study. During the maintenance treatment, patients’ CEA levels all elevated at or after the first cycle of maintenance treatment with a median CEA elevation-free survival time as 17.7 days, which was far more shorter than the median progression-free survival time evaluated by CT imaging specially for maintenance treatment (102.2 days). Before the disease progressed, the values of CEA increased steadily for several cycles with the response evaluation still as stable disease, indicating that the changes of CEA level would be earlier and more sensitive for detection of progression. The CEA kinetic was calculated with a mean of 9.6451 and a median of 8.0135, which sensitively reflected the increasing rate of CEA levels at an early stage. Our study showed that the kinetic change of CEA could be an early predictor for the progression in metastatic NSCLC patients during maintenance therapy.

## INTRODUCTION

Accounting for >80% of primary lung cancers, non-small-cell lung cancer (NSCLC) is a leading cause of cancer-related mortality all around the world [1]. Patients with advanced non-squamous NSCLC have especially poor prognosis, with only 4–6 months median progression-free survival (PFS) and 8–10 months median overall survival (OS) [2, 3]. For this group of patients, NCCN guidelines recommend bevacizumab plus chemotherapy as the preferred first-line treatment option and bevacizumab with pemetrexed as the standard maintenance treatment strategy. Although this has been proved efficacious for patients, a substantial fraction of patients were finally resistant to vascular endothelial growth factor (VEGF) based therapies within a limited duration of time, especially during the maintenance treatment [4]. In addition, the treatment response evaluation for systemic therapy is based on imaging evaluation every 2 cycles using standardized criteria, which is debated for the low sensitivity and time-lag effect [5, 6]. As a consequence, during the maintenance treatment with bevacizumab plus pemetrexed, the main challenge is to identify the disease progression at an early stage using a simple and sensitive method to optimize treatment strategy for metastatic NSCLC patients.

Carcinoembryonic antigen, first identified in 1965 by Phil Gold and Samuel O. Freedman, is related to tumor burden and therapy response [7]. Given the feasible and convenient assessment of CEA, the aim of this retrospective study was to investigate whether the kinetic change of CEA levels can be an early indicator for the progression in metastatic NSCLC patients during maintenance therapy with bevacizumab plus pemetrexed.

## RESULTS

**Figure 1:**
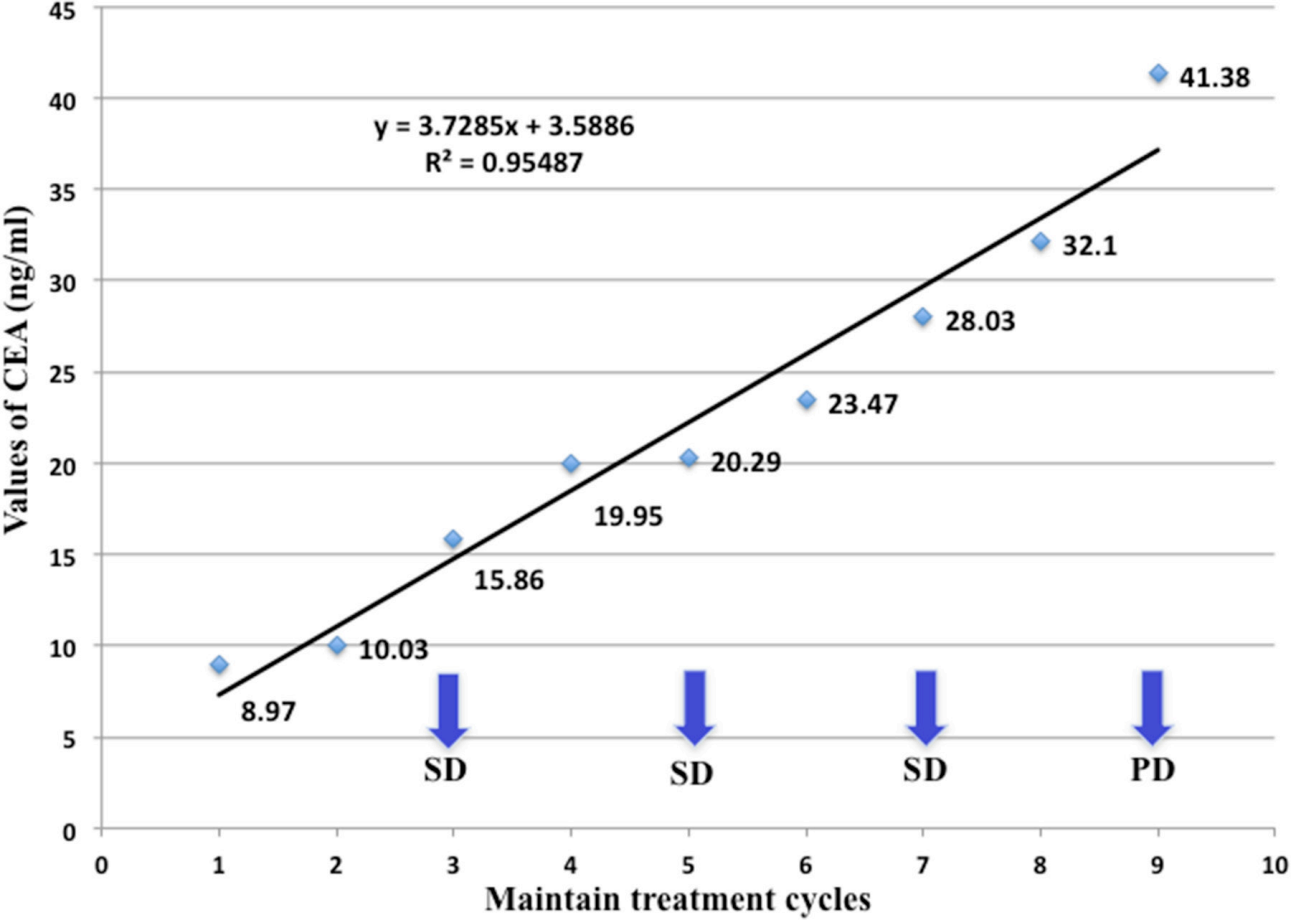
Serum CEA changes of a 44-year-old patient with metastatic *lung* adenocarcinoma during maintenance therapy including bevacizumab plus pemetrexed Her serum CEA has increased steadily from baseline 8.97 ng/mL, with all three response evaluation as SD within the 8 cycles of maintenance treatment. It was the fourth CT imaging operated before the ninth cycle of treatment discovered disease progression with the CEA level as high as 41.38 ng/mL, which reflected the changes of CEA level would be more sensitive and earlier for detection of disease progression. The kinetic of CEA level was calculated as 3.729, using the slope of a regressive curve with SPSS software.

**Figure 2:**
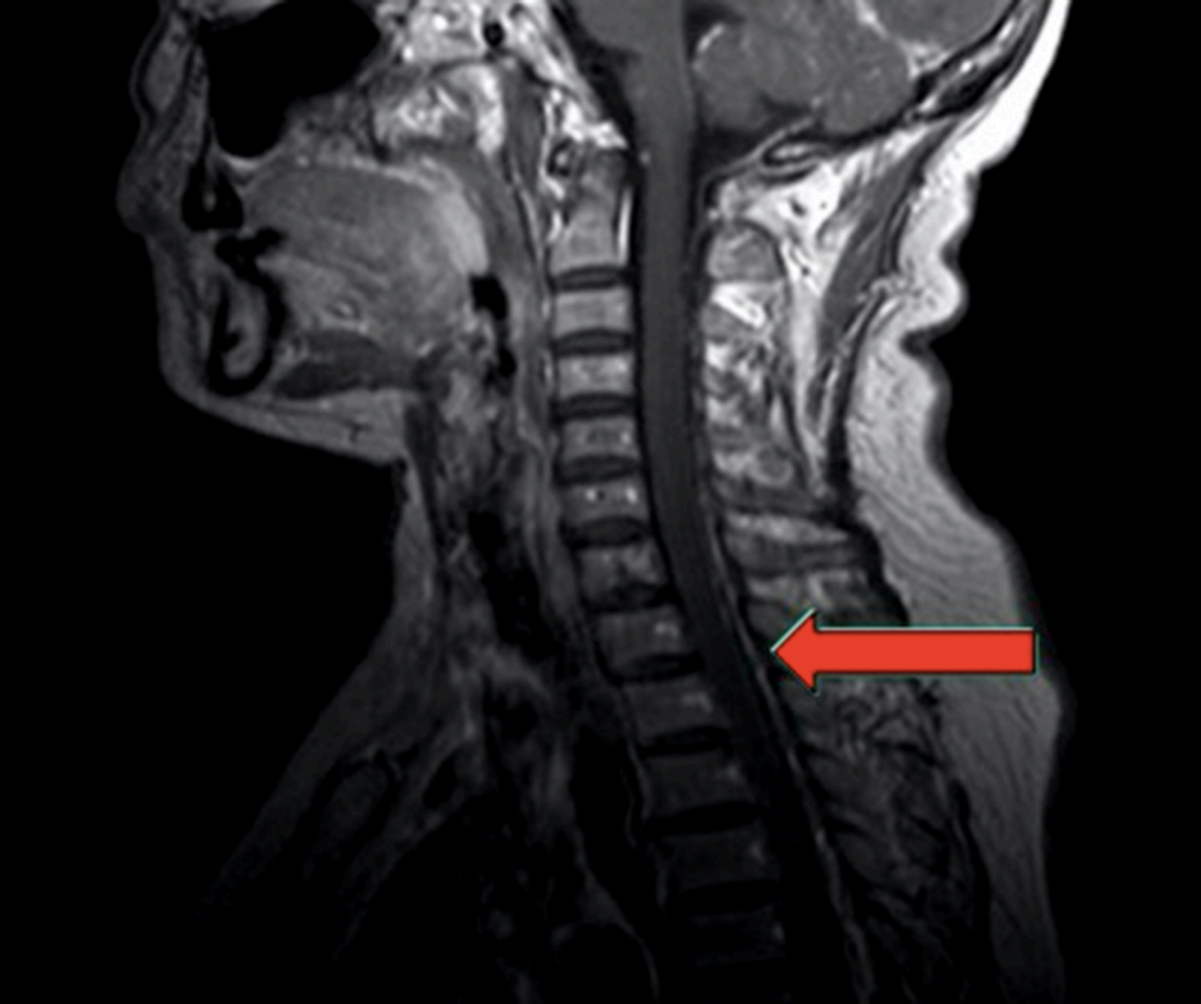
Magnetic resonance image of the same patient The image showed metastasis of spinal dura mater, which meant a failure in maintenance treatment and demand for shift of therapeutic regimen.

**Figure 3:**
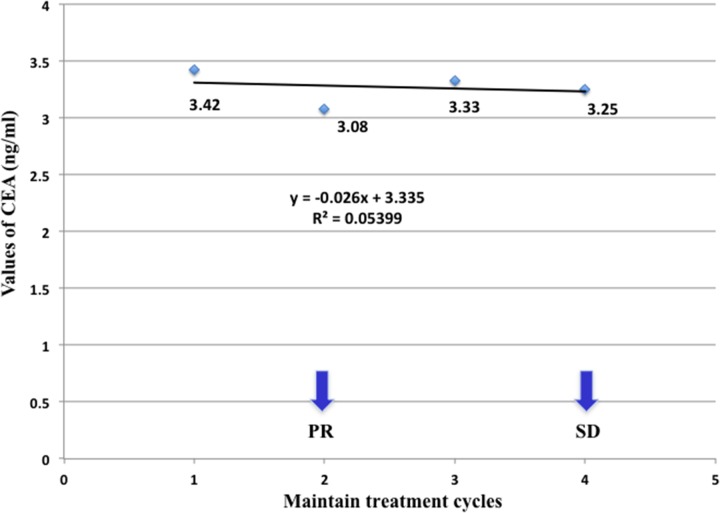
Serum CEA changes of a 73-year-old patient with metastatic *lung* adenocarcinoma during maintenance therapy including bevacizumab plus pemetrexed His serum CEA level has been stable, with the first response evaluation as PR and the second response evaluation as SD within the 4 cycles of maintenance treatment. The kinetic of CEA level was calculated as −0.026, using the slope of a regressive curve with SPSS software.

Ten patients who have received first-line treatment composed of anti-angiogenic therapy with bevacizumab plus standard chemotherapy with pemetrexed and cisplatin, and more than two cycles of sequential maintenance treatment including bevacizumab plus pemetrexed were observed (sex ratio = 3 men/ 7 women; median age, 51.3 years). With an increment varied from 0.89 to 23.77 ng/mL, patients’ CEA levels all elevated at or after the first cycle of maintenance treatment with a median CEA elevation-free survival time as 17.7 days, which was far more shorter than the median progression-free survival time evaluated by CT imaging specially for maintenance treatment (102.2 days). Before the disease progression, CEA levels of all the patients kept sustainable growth without any progressive changes in radiological evaluation according to RECIST 1.1 criteria, indicating that the changes of CEA level would be more sensitive and earlier for detection of progression. There was a 44-year-old patient whose serum CEA has increased steadily from 8.97 to 41.38 ng/mL, with all three response evaluation as SD during the 8 cycles of maintenance treatment. However, it was the fourth CT imaging operated before the ninth cycle of treatment showed that she got metastasis of spinal dura mater, which meant a failure in maintenance treatment and demand for shift of therapeutic regimen. The CEA kinetic was calculated as 3.729, which indicated a quick increasing rate of serum CEA levels (Figure 1 and Figure 2). By contrast, a stable serum CEA level usually means good treatment efficacy in clinical practice (Figure 3). In this study, the values of all patients’ CEA increased to the peak at the moment of disease progression, varied from 7.15 to 134.3 ng/mL. During the maintenance treatment, the range of CEA slopes of the regressive curves was 1.104 to 30.005, with a mean of 9.6451 and a median of 8.0135, which sensitively reflected the increasing rate of CEA levels in a kinetic way (Table 1). As a consequence of the small sample size in this study, correlations between PFS and baseline CEA or CEA kinetic was not significant.

**Table 1:** CEA kinetic changes in 10 patients during maintenance treatment

Patient no.	Cycles	Baseline CEA (ng/mL)	Firstly raised CEA (ng/mL)	CEA at progression (ng/mL)	CEA kinetic	The date for progression	The date for first CEA elevation
1	3	14.63	21.60	43.12	9.375	Day 51	Day 7
2	2	11.23	17.69	33.83	11.300	Day 56	Day 21
3	3	28.75	33.10	51.66	7.123	Day 72	Day 8
4	4	15.04	16.39	25.38	2.451	Day 119	Day 21
5	3	3.75	4.64	7.15	1.104	Day 87	Day 21
6	3	37.33	61.10	79.10	20.885	Day 117	Day 20
7	2	10.72	12.32	13.87	1.575	Day 73	Day 21
8	8	8.97	10.03	41.38	3.729	Day 244	Day 21
9	3	43.40	55.79	134.30	30.005	Day 91	Day 15
10	4	46.31	54.19	83.57	8.904	Day 112	Day 22

## DISCUSSION

Having ameliorated prognosis of patients with advanced non-squamous NSCLC, Bevacizumab, a recombinant humanized IgG1 monoclonal antibody binding with vascular endothelial growth factor, has become the focus of therapeutic intervention to block tumor angiogenesis and elevate the blood level of chemotherapeutic drug by promoting tumor vascular stabilization [8–10]. Data from the recent AVAPERL study showed a 3.7-month increase in PFS (7.4 vs. 3.7 months), which resulted in a category 2A recommendation of using bevacizumab/pemetrexed as maintenance therapy by NCCN guidelines [11]. Although this has been proved efficacious for patients, a substantial fraction of patients were finally resistant to VEGF-based maintenance therapies within a limited duration of time [4]. What’s the worse, due to the side effect of irradiation injury, the treatment response evaluation based on CT or PET-CT imaging could only be operated every 2 cycles during maintenance therapy, which is not satisfying in detecting disease progression at an early stage. Thus, with the aim to sensitively monitor disease progression, improve overall treatment results by shifting therapeutic regimen timely and reduce the cost burden of patients, continuing attempts should be made to find sensitive biomarkers that are indicative for the bevacizumab-based maintenance therapy in patients with metastatic NSCLC.

With feasible and convenient assessment methods, CEA is an important and well-known tumor biomarker for NSCLC and widely used in clinical practice. The study of Tomita. et al showed that the 5-year survival of patients with preoperative serum CEA level less and more than 2.5 ng/ml were significantly different as 79.62% and 62.0%, respectively (P = 0.0036), which indicated that a preoperative serum CEA level of ≥2.5 ng/ml was an independent prognostic factor for NSCLC patients [12]. While, Sawabata. et al used postoperative serum CEA levels as indicators for prognosis [13]. They found that the 5-year survival rate was 87% for patients with postoperative CEA levels ranged from 2.5 to 5.0 ng/mL, compared with 75% for patients with normal postoperative CEA levels (≤2.5 ng/mL) and 53% for patients with high postoperative CEA levels ≥5.0 ng/mL (P < 0.0001). In Liu. et al ’s clinical trial, they investigated the potential role of CEA in predicting response to chemotherapy and OS in patients with NSCLC and reached the conclusion that the post-treatment reduction of CEA can predict the overall response in NSCLC patients [14]. Recently, a prospective study presented that CEA ≥ 40 ng/mL was a risk factor for brain metastasis development and was associated with poor prognosis in patients with advanced NSCLC [15].

CEA has been proved to be effective in predicting the prognosis of disease. In our study, we investigated whether the kinetic change of CEA levels can be an early indicator for the progression in metastatic NSCLC patients during maintenance therapy with bevacizumab plus pemetrexed. The results of this study have showed that serum CEA levels of all the patients increased continuously before the CT imaging response evaluation as PD according to RECIST 1.1 criteria, indicating that the changes of CEA level would be more sensitive and earlier for detection of disease progression. In addition, our study was specifically evaluated the CEA kinetic by calculating of a regression line based on a minimum of three values of CEA level, which sensitively reflected the increasing rate of CEA. The baseline CEA and the CEA at disease progression of the ten patients were obviously different with great variations from 3.75 to 46.31 ng/mL and 7.15 to 134.30 ng/mL. The kinetic of CEA ranged from 1.104 to 30.005. Researches have proved that there was no significant difference between ROC curves calculated with the CEA kinetic involved in six and four CEA values for patients with disease progression or response, which meant that the CEA kinetic calculated by a few of CEA values can be useful [16]. In another way, it also indicated that the kinetic of CEA could be an early indicator for detection of disease progression during the maintenance treatment.

The mechanism for CEA values to be used as predictors for bevacizumab-based maintenance therapy may be complicated. First of all, CEA serum levels are associated with the tumor load, which would elevate when the tumor progression occurs. Furthermore, there was study observed a novel functional role of CEA in promoting endothelial cell activation and subsequent tumor angiogenesis, indicating that increasing CEA can antagonize the anti-angiogenesis effect of bevacizumab and be a cause for the failure of the bevacizumab-based maintenance therapy [17]. In addition, CEA has been shown to inhibit tumor cell anoikis by preventing apoptosis upon cell detachment and by interfering with cell differentiation [18, 19]. Therefore, It was reasonable to use CEA values to predict treatment response in anti-VEGF-based therapies.

In conclusion, with the main advantages of CEA values, namely, low cost, convenient detection, and accessibility of using a standard personal computer to calculate the sensitive CEA kinetic, the serum CEA levels should be observed during the bevacizumab-based maintenance treatment in patients with metastatic NSCLC to predict early disease progression and subsequently to optimize and individualize the treatment by modifying the therapeutic regimen. Further studies included larger numbers of patients are expected to validate such a finding and a cut-off value of CEA kinetic should be defined for disease progression. In addition, there is now extensive research on imaging response markers that may be better indicators than traditional RECIST criteria [20, 21]. In the future it could be worthwhile to combine circulating biomarkers and newer imaging markers for more reliable response assessment.

## MATERIALS AND METHODS

### Patients

Ten patients with metastatic lung adenocarcinoma treated at our hospital with a first-line therapy including bevacizumab-based chemotherapy and a following maintenance therapy including bevacizumab plus pemetrexed from June 2015 to October 2016 were recruited in this study, all of whom met the eligibility criteria: ≥18 years old; histologically confirmed lung adenocarcinoma; unresectable metastasis; an Eastern Cooperative Oncology Group performance status <2; and adequate renal and hepatic function. The patients from our hospital received first-line therapy composed of anti-angiogenic therapy with bevacizumab (7.5 mg/kg every 21 days) plus standard chemotherapy with pemetrexed (500 mg/m^2^ every 21 days) and cisplatin (75 mg/m^2^). After four or six cycles of treatment, all the patients got partial response or stable disease according to the revised Response Evaluation Criteria in Solid Tumors (RECIST) 1.1 criteria and received the subsequent maintenance treatment including bevacizumab (7.5 mg/kg every 21 days) plus pemetrexed (500 mg/m^2^ every 21 days). This cohort was analyzed to assess the predictive value of CEA for metastatic NSCLC patients during bevacizumab-based maintenance treatment. Table 2 summarizes the clinical characteristics of the patients. This study was approved by the Shandong Cancer Hospital Institutional Review Board.

**Table 2:** Clinical characteristics of patients

Patient no.	Gender	Age	ECOG score	Gene type	Stages	Metastatic locations	First-line regimen	Cycles	Response evaluation
1	F	52	1	Wild type	cT4N3M1	Brain, liver	PP+Bev	4	SD
2	F	59	1	Wild type	cT4N1M1	Pleura	PP+Bev	4	SD
3	F	61	1	Wild type	cT2N3M1	Pleura	PP+Bev	6	PR
4	F	48	1	Wild type	cT2N3M1	Brain	PP+Bev	4	SD
5	M	43	0	EGFR mut	cT1N3M1	Bone	PP+Bev	4	SD
6	M	55	1	EGFR mut	cT2N3M1	Bone	PP+Bev	4	PR
7	M	59	0	Wild type	cT4N3M1	Adrenal gland	PP+Bev	4	SD
8	F	44	1	Wild type	cT2N2M1	Brain	PP+Bev	6	SD
9	F	41	0	Wild type	cT2N3M1	Bone	PP+Bev	4	SD
10	F	51	1	EGFR mut	cT2N1M1	Liver	PP+Bev	6	SD

M, male; F, female; ECOG, Eastern Cooperative Oncology Group; EGFR, epidermal growth factor receptor; PP, pemetrexed plus platinum; Bev, bevacizumab; PR, partial response; SD, stable disease.

### Carcinoembryonic antigen level assessment

Serum CEA levels of patients with metastatic lung adenocarcinoma were centrally determined at day 1 for each cycle of bevacizumab-based maintenance treatment. The CEA serum levels were measured with commercial electrochemiluminescence immunoassay using Elecsys cobas e601 analyzer and reagent kits (Roche Diagnostics, Mannheim, Germany) and results were given as ng/mL. The kinetic of CEA level was calculated based on at least three values of serum CEA, using the slope of a regressive curve with SPSS software.

### Assessment of response

Assessment of response was determined every two cycles according to the revised Response Evaluation Criteria in Solid Tumors (RECIST) 1.1 criteria: a complete response (CR) was defined as the disappearance of all clinical target lesions for at least 4 weeks without no lesion appearing, a partial response (PR) was defined as a 30% or greater decrease in the sum of the longest diameters of target lesions taking the sum at baseline as the reference, progressive disease (PD) was defined as an increase of at least 20% in the sum of the greatest dimensions of treated lesions or the appearance of one or more new lesions, and stable disease (SD) was defined as neither sufficient shrinkage to qualify as a PR nor a sufficient increase to qualify as PD [22].
