# Correction: Kinetic change of serum carcinoembryonic antigen can early predict progression in patients with metastatic non-small cell lung cancer during maintenance therapy with bevacizumab plus pemetrexed

**DOI:** 10.18632/oncotarget.23291

**Published:** 2017-12-15

**Authors:** Nasha Zhang, Li Kong, Fang Shi, Wang Jing, Haiyong Wang, Ming Yang, Jinming Yu, Hui Zhu

**Affiliations:** ^1^ Cheeloo College of Medicine, Shandong University, Jinan, China; ^2^ Department of Radiation Oncology, Shandong Cancer Hospital and Institute, Shandong Cancer Hospital Affiliated to Shandong University, Jinan, China; ^3^ Shandong Academy of Medical Sciences, Jinan, China

This article has been corrected: the authors first and last names order was updated.

Original article: Oncotarget. 2017; 8:74910-74916. https://doi.org/10.18632/oncotarget.20456

